# A patient with testicular pseudolymphoma – a rare condition mimicking malignancy: a case report

**DOI:** 10.1186/1752-1947-1-71

**Published:** 2007-08-25

**Authors:** Roman Ganzer, Maximilian Burger, Matthias Woenckhaus, Wolf Ferdinand Wieland, Andreas Blana

**Affiliations:** 1 Department of Urology, University of Regensburg, St Josef's Hospital, Landshuter Straße 65, 93053 Regensburg, Germany; 2 Department of Pathology, University of Regensburg, Franz – Josef – Strauß Allee, 93053 Regensburg, Germany

## Abstract

Three months following a right sided acute epididymitis a 62 year old patient presented with a painless right testicular swelling. Physical examination, scrotal ultrasound and operative exploration suggested malignancy. However, after inguinal orchiectomy a benign pseudolymphoma of the testis was revealed by pathological examination. A pseudolymphoma is a rare benign lesion which can only be distinguished from a malignant lymphoma by immuno-histochemistry and molecular-genetical investigation techniques.

## Background

Testicular cancer presents in 5% of all urological tumours with 3 to 6 new cases occurring per 100000 per year [1]. Testicular germ cell tumours have a peak incidence in the 3^rd ^and 4^th ^decade, but also occur in the elderly. Testicular cancer generally appears as a painless intrascrotal mass and is usually diagnosed by physical examination and scrotal ultrasound. In case of a suspected testicular mass the patient must undergo inguinal exploration. If a tumour is found, orchiectomy with resection of the spermatic cord is performed and the specimen sent for pathological examination. Benign lesions of the testis which appear to be malignant on physical examination and scrotal ultrasound are rare. We describe the case of a patient who underwent inguinal orchiectomy of a suspected malignant tumour, which finally showed to be a pseudolymphoma of the testis, a rare benign lesion mimicking features of malignancy.

## Case presentation

**Figure 1:**
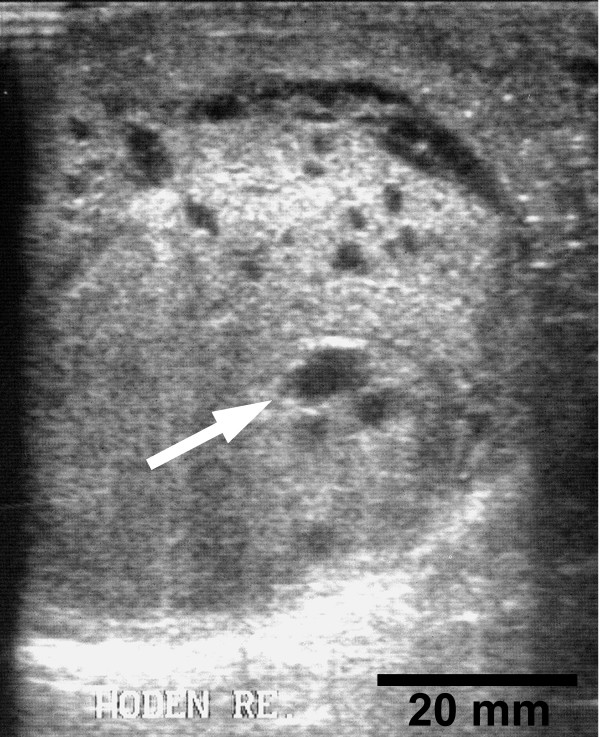
Ultrasonography, 8.0 MHz. Sagittal section of right testis showing areas of inhomogeneous parenchyma and cystic lesions (arrowhead).

A 62 year old male patient was referred to our department with a right sided acute epididymitis which was cured without complications by antibiotics. Three months later the same patient presented again with a painless indurated swelling of the right testis which he had noticed incidentally. Physical examination of the external genitalia showed an enlarged indolent right testis with multiple inhomogeneous indurations. Scrotal ultrasound revealed an enlarged right testis with areas of inhomogeneous parenchyma and cystic lesions (Fig. 1). Physical examination, blood tests and urinalysis showed no signs of inflammation. Alpha – fetoprotein (AFP), chorionic gonadotrophin (β-HCG) and lactate dehydrogenase (LDH) were within normal limits. A right testicular exploration by inguinal approach was performed to exclude a malignant testicular tumour. Due to the intraoperative aspect of malignancy (inhomogeneous enlargement with indurated and cystic areas and involvement of the tunica vaginalis testis) the testis was removed by high inguinal semicastration. However, final histology report revealed a severe combined chronic epididymitis and orchitis with destructive infiltrates of lymphocytes of polyclonal type with the formation of a testicular pseudolymphoma. The tunica vaginalis was transformed by inflammatory pseudocysts and nodular regions of granulomatous tissue. No evidence of malignancy could be demonstrated by various investigation techniques including histomorphology, immuno – histochemistry and molecular genetic studies.

## Discussion

A history of painless testicular swelling in combination with inhomogeneous parenchyma on ultrasound beside the intraoperative aspect described above is highly suspicious for testicular cancer. Even in the case of not elevated tumour markers a classical seminoma or a non germ – cell tumour including malignant lymphoma may be present. Therefore, surgery was indicated in this case and a wait and see strategy would not have been justifiable. However, our case demonstrates that in rare conditions chronic inflammatory pseudotumours may mimic typical presentations of testicular malignancy and particularly in post inflammatory conditions have to be included in the differential diagnosis.

**Figure 2:**
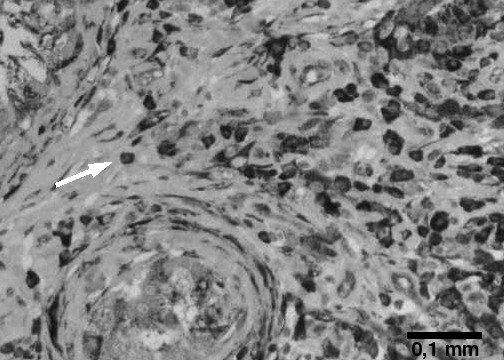
Testis, immuno – histochemical staining for the plasma cell marker Vs38c showing diffuse infiltration of testicular tissue by plasma cells (arrowhead).

A pseudolymphoma is a reactive benign lesion a few cases of which have been reported in the skin [2], the GI – tract [3], the lung [4], in Sjögren's syndrome [5] and once in the kidney [6]. Histological investigation techniques are not capable of distinguishing a pseudolymphoma from a malignant lymphoma and therefore have to be supplemented by immuno – histochemistry (Fig. 2) and molecular – genetical methods to exclude monoclonal lymphocyte proliferation [7]. To our best knowledge, only five cases of testicular manifestation of a pseudolymphoma have been described in the literature before [8]. As the condition is benign the patient was cured by surgery and no further staging investigations were necessary.

## Conclusion

A pseudolymphoma of the testis is a rare condition mimicking malignancy which occurs after chronic inflammation and has to be included in the differential diagnosis of painless testicular swelling.

## Competing interests

The author(s) declare that they have no competing interests.

## Authors' contributions

All authors have made substantial contributions to concept this case report
